# Closure of a Giant Saphenous Vein Graft Aneurysm with Embolization Coil

**DOI:** 10.4061/2009/748272

**Published:** 2009-10-11

**Authors:** Ashwani Kumar, Dixon Santana, Leigh Ann Jenkins

**Affiliations:** ^1^Division of Cardiology, Department of Medicine, Texas Tech University Health Sciences Center, 3601 4th Street, Lubbock, TX 79430, USA; ^2^Division of Vascular Surgery, Department of Surgery, Texas Tech University Health Sciences Center, 3601 4th Street, Lubbock, TX 79430, USA

## Abstract

Aneurysms of saphenous vein grafts (SVGs) to coronary arteries are rare, usually asymptomatic and found incidentally. We report a case of an 84-year-old female who was found to have 8.1 × 8.4 cm aneurysm of an SVG to obtuse marginal (OM) artery. The aneurysm was prior to the distal anastamosis but no flow into the OM artery was noted. Cook Tornado Embolization Coils were used successfully to occlude the SVG proximal to the aneurysm. No complications occurred. The use of embolization coils is an effective and safe method for aneurysm occlusion when the anatomy is suitable and especially when patient is high risk for repeat surgical intervention.

## 1. Introduction

Aneurysms of aortocoronary saphenous bypass grafts (SVGs) are an unusual complication. Mild dilation of SVG is not uncommon and has been reported to occur in 14% of cases after 5 to 7 years following surgery [1]. Large aneurysms of SVG are rare and were first reported by Riahi et al. [2]. Clinical presentation may vary from an asymptomatic patient, recurrent chest pain, myocardial infarction, and rupture or sudden cardiac death [3, 4].

 The diagnosis should be considered in all patients presenting with a hilar or mediastinal mass following aortocoronary bypass (ACB), as timely treatment may avert aneurysm rupture and death. There is no standard method to treat these patients. We report a case of a large aneurysm of SVG to obtuse marginal artery which was successfully treated with percutaneous coil embolization of the SVG.

## 2. Case Report

**Figure 1:**
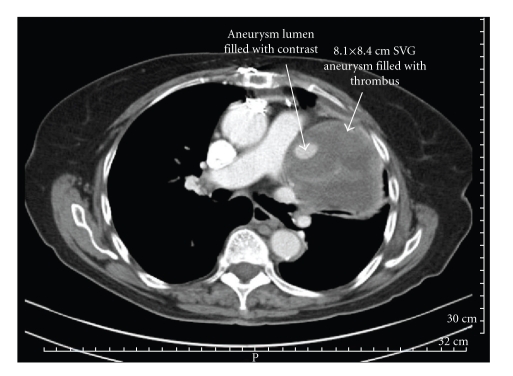
CT scan chest. An 8.1 × 8.4 cm mass is noted adjacent to left ventricle. Central filling (2 × 2 cm) of the mass with contrast is seen and is surrounded by thrombus.

**Figure 2:**
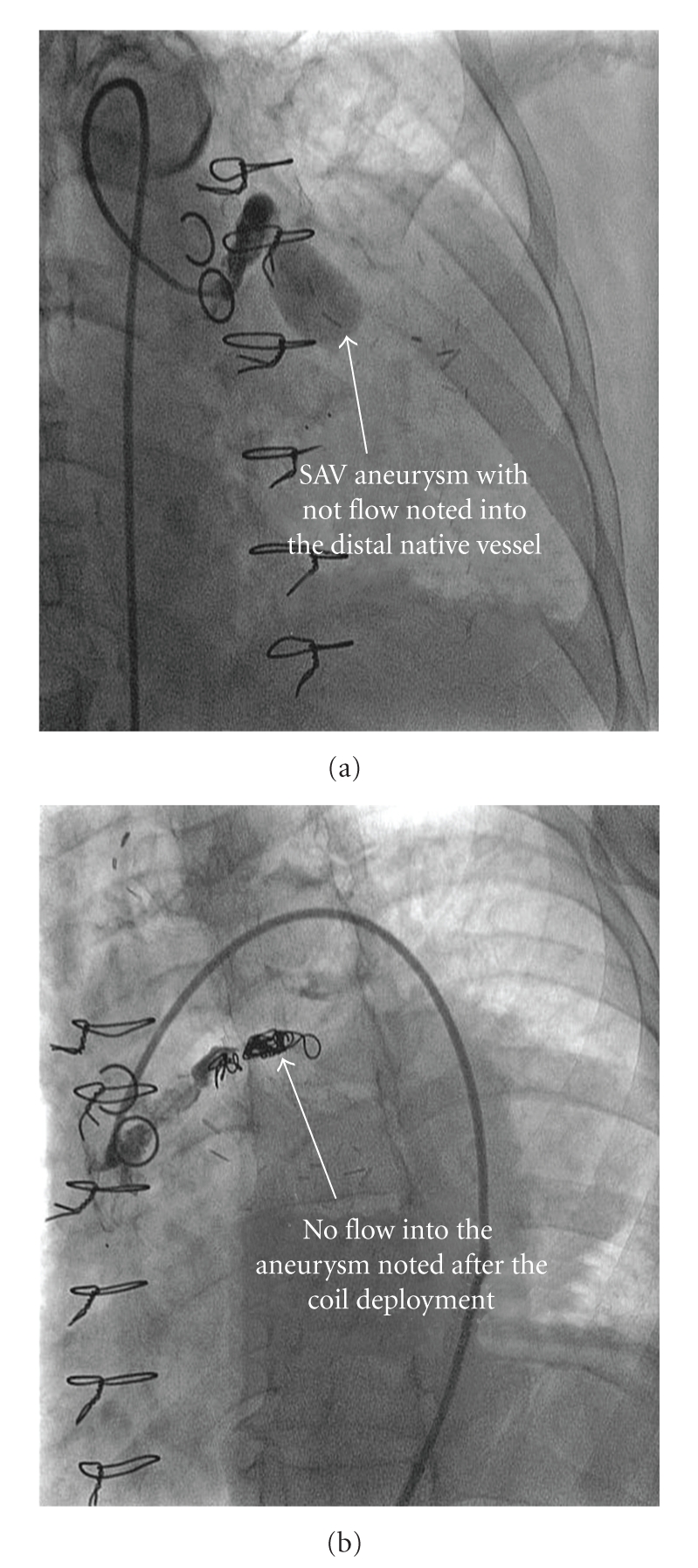
Saphenous vein graft angiogram, demonstrates the flow into the aneurysm before (a) SVG aneurysm with no flow noted into the distal native vessel. (b) the coil embolization.

An 84-year-old female with hypertension and hyperlipidemia had had two vessel coronary ACB 18 years ago with an SVG to left anterior descending artery (LAD) and obtuse marginal artery (OM). Patient had redo ACB 8 years ago with a left internal mammary artery (LIMA) to LAD, and SVG to OM, as she was found to have diffusely diseased SVG to LAD and a totally occluded SVG to OM. Recently, she was found to have a mediastinal mass on routine chest X ray which was done for evaluation of cough and fever. Subsequently, CT scan of chest with contrast was done which revealed a large heterogeneous mass measuring 8.1 × 8.4 cm in size, located on left side of the heart with active flow and large clot burden within the mass (Figure 1). The coronary angiogram demonstrated a patent LIMA to LAD and patent SVG to OM. A 2 × 3 cm aneurysm was found on injection of another SVG, presumed to be the old SVG to OM, without any distal flow into any native vessel (Figure 2). The length of the SVG from aorta to aneurysm was 4 cm which was diffusely diseased. A diagnosis of aneurysm of SVG was made. The discrepancy in the aneurysm size between CT scan and angiogram highlights the importance of CT scan in assessing the exact size of aneurysm.

 The decision was made to occlude flow to the aneurysm due to the risk of its rupture, which is associated with high mortality and morbidity. The SVG feeding the aneurysm was selectively engaged with a 7 French JR4 guide catheter. A standard 0.35 inch Glide wire (Terumo Medical Corporation) was advanced into the aneurysm over which a 5 French Glide catheter was placed at the origin of the aneurysm. The glide wire was removed and an 8 × 5 mm Cook Tornado Embolization coil was deployed. A total of five coils were deployed more proximally in the SVG (Figure 2). Angiography demonstrated complete cessation of flow into the aneurysm (Figure 2). The patient was discharged the next day without any complications.

## 3. Discussion

Minor SVG aneurysmal dilation is common, reported up to 14% at 5–7 years after ACB [1]. Giant SVG aneurysm is defined as being greater than 4 cm in diameter [5] and is a rare occurrence after ACB. The true incidence may be underestimated as many patients are asymptomatic before a fatal cardiac event.

 The true aneurysm usually develops in the body of the graft and is the result of a chronic degenerative process caused by vascular injury from hyperlipidemia and progression of atherosclerosis. Valve insertion sites are especially prone to develop aneurysms where smooth muscle in the media changes from circular to a weaker longitudinal orientation. Other possible contributing factors include varicosities in the vein graft and vascular injuries from surgical trauma. The pseudoaneurysms are typically located at the site of proximal anastomosis. They are thought to occur because of tension at the site of anastomosis or from technical issues with suture placement. Infection is commonly associated with suture line dehiscence and pseudoaneurysm formation.

 The clinical presentation of SVG aneurysm is highly variable but typically presents several years after the surgery as an incidental finding of a mediastinal mass on chest imaging in otherwise asymptomatic individuals. SVG pseudoanuerysms tend to present much earlier than the true aneurysms [6], the distinction between true and false aneurysm can be difficult to determine preoperatively. Other presentations include recurrent chest pain, infection, hemoptysis, hemothorax, hemopericardium, and sudden cardiac death [6]. Untreated aneurysms have caused myocardial infarction [3], fistula [7], rupture [4], and death. The exact incidence of rupture is difficult to assess due to rarity of this condition, but rate of rupture appears to be high with larger and pseudoaneurysm [4]. Timely management of these patients can prevent these complications.

 An SVG aneurysm must be considered in every case of hilar or mediastinal mass noted in a patient with prior ACB [8]. Evaluation of any suspected SVG aneurysm should include CT scan which can demonstrate flow in the aneurysm and delineate anatomic relationships of the aneurysm with other structures. Coronary angiography should be performed to evaluate graft patency as well as define other coronary lesions that should be addressed.

 The optimal management of SVG aneurysm remains controversial. Traditionally, repeat ACB and aneurysm resection has been advised for large aneurysms. It is important to note that repeat ACB carries significant risk of mortality in elderly patients and pseudoanuerysm recurrence has been reported at the site of aortic anastomosis despite corrective surgery [5]. Conservative medical therapy with chronic anticoagulation appears ineffective in preventing anuerysmal dilation or in situ thromboembolism [5]. Percutaneous methods should be considered first to treat the aneurysm especially in elderly patients where risk of repeat surgery is very high.

 Various percutaneous methods have been used in the treatment of SVG aneurysm, depending upon the underlying anatomical properties of the aneurysm and coronary circulation. Polytetrafluoroethylene covered stents appear to be useful when continued antegrade SVG flow is desired. If the distal graft is occluded or the coronary territory is perfused, graft occlusion may be considered with thrombin injection [9], vascular plug [10], or coil embolization.

 Because of the variable nature aneurysms and the status of distal coronary circulation, the ideal method will continue to be determined on a case-by-case basis given the rarity of this condition.

## References

[1] Memon A-Q, Huang RI, Marcus F, Xavier L, Alpert J (2003). Saphenous vein graft aneurysm: case report and review. *Cardiology in Review*.

[2] Riahi M, Vasu M, Tomatis LA (1975). Aneurysm of saphenous vein bypass graft to coronary artery. *Journal of Thoracic and Cardiovascular Surgery*.

[3] Forster DA, Haupert MS (1991). Large mediastinal mass secondary to an aortocoronary saphenous vein bypass graft aneurysm. *Annals of Thoracic Surgery*.

[4] Wester DJ, Martinez HO, Camp A (1993). Aneurysm of a saphenous vein graft manifested as a mediastinal mass on chest radiographs. *American Journal of Roentgenology*.

[5] Topaz O (2006). Giant aneurysms of saphenous vein grafts: management dilemmas and treatment options. *Catheterization and Cardiovascular Interventions*.

[6] Puri R, Dundon BK, Leong DP, Worthley SG, Worthley MI (2009). Giant saphenous vein graft pseudoaneurysm rupture presenting with cardiac tamponade. *Heart, Lung and Circulation*.

[7] Williams ML, Rampersaud E, Wolfe WG (2004). A man with saphenous vein graft aneurysms after bypass surgery. *Annals of Thoracic Surgery*.

[8] Almanaseer Y, Rosman HS, Kazmouz G, Giraldo AA, Martin J (2005). Severe dilatation of saphenous vein grafts: a late complication of coronary surgery in which the diagnosis is suggested by chest X-ray. *Cardiology*.

[9] Tamirisa PK, Rinder M, Singh J, Halle A, Lasala J (2002). Thrombin injection to treat pseudoaneurysm of internal mammary artery bypass graft: a case report. *Catheterization and Cardiovascular Interventions*.

[10] Mylonas I, Sakata Y, Salinger MH, Feldman T (2006). Successful closure of a giant true saphenous vein graft aneurysm using the Amplatzer vascular plug. *Catheterization and Cardiovascular Interventions*.

